# Utility of eButton images for identifying food preparation behaviors and meal-related tasks in adolescents

**DOI:** 10.1186/s12937-018-0341-2

**Published:** 2018-02-24

**Authors:** Margaret Raber, Monika Patterson, Wenyan Jia, Mingui Sun, Tom Baranowski

**Affiliations:** 1 Department of Pediatrics Research, University of Texas MD Anderson Cancer Center, Houston, USA; 2 USDA/ARS Children’s Nutrition Research Center, Baylor College of Medicine, Houston, USA; 3 Department of Neurological Surgery, University of Pittsburg, Pittsburg, USA

**Keywords:** Food preparation, Nutritional assessment, All-day imaging, Cooking, Adolescents

## Abstract

**Background:**

Food preparation skills may encourage healthy eating. Traditional assessment of child food preparation employs self- or parent proxy-reporting methods, which are prone to error. The eButton is a wearable all-day camera that has promise as an objective, passive method for measuring child food preparation practices.

**Purpose:**

This paper explores the feasibility of the eButton to reliably capture home food preparation behaviors and practices in a sample of pre- and early adolescents (ages 9 to 13).

**Methods:**

This is a secondary analysis of two eButton pilot projects evaluating the dietary intake of pre- and early adolescents in or around Houston, Texas. Food preparation behaviors were coded into seven major categories including: browsing, altering food/adding seasoning, food media, meal related tasks, prep work, cooking and observing. Inter-coder reliability was measured using Cohen’s kappa and percent agreement.

**Results:**

Analysis was completed on data for 31 participants. The most common activity was browsing in the pantry or fridge. Few participants demonstrated any food preparation work beyond unwrapping of food packages and combining two or more ingredients; actual cutting or measuring of foods were rare.

**Conclusions:**

Although previous research suggests children who “help” prepare meals may obtain some dietary benefit, accurate assessment tools of food preparation behavior are lacking. The eButton offers a feasible approach to food preparation behavior measurement among pre- and early adolescents. Follow up research exploring the validity of this method in a larger sample, and comparisons between cooking behavior and dietary intake are needed.

## Background

Diet is a modifiable risk factor for several chronic diseases including heart disease, obesity and cancer [1–3]. Child and adolescent-targeted prevention efforts are vital to establishing healthy dietary habits early in life [4, 5]. Understanding child eating habits is critical for the design and evaluation of nutrition education programs, however, measurement of dietary intake and related factors has proven challenging [6]. Traditionally, self- or parent proxy-reported methods have been used to measure child and adolescent dietary intakes [7], food preparation skills and practices [8], knowledge [9] and preferences [8, 9]. These assessment tools, while widely used, have demonstrated modest validity when compared to observation and biomarker verification [10–12]. Novel technologies such as all day cameras and other wearable devices have been utilized to develop more sensitive lifestyle assessment tools for children [13–15]. All day cameras show promise as a passive mechanism for measuring child diet [15] and may also provide valuable information regarding food preparation practices.

Home cooked meals are generally lower in saturated fat, sugar and calories and higher in fruit and vegetables compared to foods eaten out of the home [16–18]. Increased frequency of home cooked meals has been associated with better healthy eating index (HEI-2010) scores [19] and lower meal costs [19], making food preparation skill development an attractive target for healthy diet promotion.

Frequent family meals may promote healthier diets, emotional well-being and lower weight status of children [20–22]. The benefits of family meals and home cooking may be amplified by the inclusion of children in food preparation. For example, adolescent involvement in meal preparation has been associated with increased fruit, vegetable, iron, calcium, vitamins D and C, and folate intake, however these trends were not detected when parents prepared meals without their child’s assistance [23]. Similarly promising effects have been observed in younger children [24–26].

Programs to promote food preparation skills among youth have gained popularity worldwide [27–30]. Youth cooking programs, however, are extremely variable in curriculum content and evaluation approach [8]. Attempts to measure cooking skills tend to assess simple cooking frequency [31] or cooking of specific items (e.g. ability to bake a cake) [32]. Youth participation in food preparation is typically gauged through self- or parent-reported items that focus on frequency of assistance [23, 33]. More objective measures may illuminate the details of how youth engage in food preparation, which is currently absent in extant self-report tools. This deeper understanding of youth cooking behavior may in turn support practical nutrition intervention development and evaluation.

The eButton is a wearable electronic device for passive assessment (i.e. requires no volitional effort to initiate assessment once the device has been successfully turned on) of diet, physical activity and lifestyle behaviors for both adults and children. It uses a small camera located on the chest to take images at four-second intervals throughout the day [34]. Analysis of these images has been used to objectively assess children’s dietary intake [15]. The collection of passive, observational images of child food preparation activities offers promise as a more precise measure of child cooking behaviors. This paper explores the ability of eButton image analysis to capture home food preparation behaviors/practices in a sample of pre- and early adolescents. A secondary aim was to describe the meal and food preparation habits observed in the sample.

## Methods

This study employed observational methods and is presented as per STROBE (a checklist to Strengthen the Reporting of Observational Studies in Epidemiology) research guidelines [35].

### Participants

Data from two eButton pilot projects evaluating dietary intake of pre- and early adolescents who lived in or around Houston, Texas were re-analyzed for food preparation and meal-related tasks. The recruitment strategies were comparable between the two studies. Child-parent dyads were recruited by phone from an institutional volunteer participant database, flyers posted across the Texas Medical Center campus, and online announcements promoting the study. Inclusion criteria for the first study included: children aged 8–13, who spoke and read English and were willing to complete all measures. Further, parents needed to give permission for children to wear the eButton, be willing to answer questions about the eButton and have sufficient Internet access at home to download resulting data [15]. Inclusion criteria were similar for the second study, except that only children aged 9–13 were recruited. Ten participants were recruited for the first pilot study, and 31 participants recruited for the second. Although 8 year olds were eligible for the first study, none were included in this analysis.

### eButton description and procedures

The eButton is a multi-sensor device that attaches around the collar, to the shirt of a participant and includes a camera, a 9-axis motion sensor (including an accelerometer, a gyroscope and a magnetometer, each providing three-dimensional measurements), a barometer, a temperature sensor, and a light sensor. It also includes data storage on a MiniSD flash card and a lithium-ion battery. The camera recorded pictures of everything in front of the wearer at four-second intervals throughout the wearing period. The images were automatically encrypted upon taking and therefore safely sent via email from participants. Upon retrieval, the images were de-encrypted and de-identified (visible faces blurred using specialized software) by study staff in preparation for analysis [14, 15, 34].

Children and parents were given a full explanation of the eButton device and detailed instructions for its use. Participants in the first study were instructed to wear the eButton for one day only as this was an early pilot study of the eButton in children. Participants in the second study were instructed to wear the device for two days. Participants were encouraged to wear the device from waking until bed-time, including at school if during a weekday. Participants were given a letter for their school principals that explained the study and eButton device. Only one day per participant was used in this analysis for consistency. Parents emailed encrypted image files to research staff at the end of each day. Participants also kept a log of any software issues they encountered while using the device [15].

### Measurement tools

The eButton hardware was complemented by software for data processing, including one for dietary intake assessment and another focused on activity categorization. The activity categorization software used accelerometer data to divide the images into homogenous events, allowing researchers to view the photos in clusters of common activity [14]. This project utilized the activity categorization software to identify food preparation events. One day of images for each participant was viewed in its entirety to ensure all food preparation events were analyzed.

Food preparation behaviors identified in the images were encompassed by seven major categories (Table 1) including browsing, altering food on plate/adding seasoning, food media, tasks, prep work, observing and cooking. “Browsing” specifies moments when a participant is facing food storage, either in the fridge, freezer, pantry, on the kitchen counter, at the grocery store, at school in the lunch line or at a convenience store or café. The browsing subcategories focus on where participants were most likely to be exposed to food options. This information is pertinent to initiatives to improve healthy food choices, improve food labeling, or restrict high sugar/high calorie food in the home and at school [36–38]

**Table 1:** Description of activity categories used during analysis

Activity category	Subcategory	Description
Browsing: Any apparent moments when the child is actively looking at or for food in various locations. Does not include just walking through kitchen without pausing.	Pantry	Standing in front of open unit containing dry stored foodstuffs. May be a shelf, walk in pantry, or extra pantry (in garage for example)
	Fridge	Standing in front of open fridge or freezer
	Grocery Store	Walking through grocery store and/or looking at items. Includes most types of food markets and specialty food shops, does not include convenience store or gas station
	Convenience Store or Café Counter	Walking through and/or looking at items in convenience store or café including: gas station, corner store, ice cream shop display, coffee shop display, other sorts of retailers with display cases of food to select. Does not include sit-down restaurants.
	Kitchen Counter	Standing in front of counter space containing food (i.e.: bowl of fruit on counter)
	At School	Walking through and/or looking at items in school lunch line
Altering Food on Plate/sAdding Seasoning: Making any alterations to food already plated in front of child such as adding salt or other seasonings, hot sauce, ketchup or other condiments. Also includes removing elements of already plated food such as taking crusts off of bread or removing candies from cereal. Does not include seasoning food while cooking.		
Food Media, Recipes, Cookbooks, Photo: Child takes photo of food using any camera device, reads or looks at cookbooks/recipes, visits food/cooking related websites/apps or appears to be watching a cooking-related tv show		
Meal Related Tasks: Activities related to meal behaviors that do not include working with food directly	Plating	Moving food from cooking or serving vessels to one’s own or another’s plate. Includes getting second servings. Does not include a parent or peer putting food onto plate in front of the child.
	Getting Drinks	Pouring beverage into a cup for oneself or for another. Includes getting up to get the beverage from the kitchen or pouring from a container on the table/in the eating space. Includes retrieving a squeeze pack or bottled drink. Does not include a parent or peer pouring a drink for the child.
	Clearing and/or washing dishes	Moving used dishes from table or eating space to the kitchen, either directly to the sink or dishwasher or to the counter. Also includes rinsing dishes, washing dishes, and/or moving dishes into the dishwasher as well as wiping down the sink space. Includes putting clean dishes away.
	Setting the Table	Placing napkins, plates, cutlery, serving vessels or other necessary dining accessories on a table or in eating space.
Prep Work: Includes all preparation of food for eating or cooking. Does not include heating of any food items.	Washing and/or Peeling Produce	Includes rinsing of all types of fruits and vegetables with water. Also includes peeling produce by hand (i.e. orange) or by peeler (i.e. potatoes).
	Cutting	Includes all cutting of food products with knives regardless of size or apparent sharpness. Also includes use of graters/mandolins or other sharp cutting equipment.
	Cracking Eggs	Cracking eggs by hand. Does not include any other activity.
	Combining 2+ ingredients by hand	Combining of two or more ingredients by hand in a range of settings including preparing cereal (combination of cereal and milk), making sandwiches (combination of bread and other products), mixing together a salad, and all general mixing of ingredients by hand. Includes mixing beverages such as chocolate sauce/powder with milk or hot tea with honey. Does not include placing ingredients in small appliance.
	Measuring	Demonstrated use of measuring tools including measuring cups, spoons and liquid measures. Does not include rough estimates or “eyeballing”.
	Unwrapping	Removing packaging from foods. Includes all snacks such as chips, granola bars, ice cream bars etc. Also includes unwrapping raw packaged meat, fruit/vegetables, cheese and other staples before preparation.
	Using Blender/Small appliance	Placing items in kitchen stand mixer, in bowl fitted with electric mixer, blender, or food processor. Does not include using a small cooking appliance such as a counter grill or electric kettle.
Cooking: Any direct use of heat-based cooking methods.	Stove	Includes any cooking done on stove top burners including boiling water or helping adult cooking, also includes cooking on stove independently. Does not include using small cooking appliances.
	Oven (Baking)	Includes any cooking done in oven including baking of cookies/cakes etc., reheating of left-overs or frozen snacks, and any other baking/roasting/broiling.
	Microwave	Includes any use of microwave for food such as heating water/milk, making microwave popcorn, reheating pre-prepared foods, defrosting foods or cooking foods fully in microwave.
Observing: Any apparent witnessing of food / meal behaviors by adults and/or peers. Does not include adults and/or peers simply being present in kitchen/near food. Does not include observing consumption.	Food Selection by Adults	Witnessing any adults in household select foods to consume or prepare from home. Includes selection from pantry, fridge, counter and stores.
	Food Selection by Peers	Witnessing any child (not apparent adult) select foods to consume or prepare from home. Includes selection from pantry, fridge, counter, stores and school.
	Any Food Preparation by Adults	Any food preparation or cooking undertaken by adults including combining ingredients, using appliances, using heat-application methods. Also includes unwrapping of food products as well as heating of pre-prepared meals and plating of foods. Does not include consumption.
	Any Food Preparation by Peers	Any food preparation or cooking undertaken by children (not adults) including combining ingredients, using appliances, using heat-application methods. Also includes unwrapping of food as well as heating of pre-prepared meals and plating of foods. Does not include consumption.

.

“Altering food or adding seasoning” specifies the actions adolescents took to subtly alter foods once they were plated, mainly through seasoning or condiment use. This behavior could occur at home or outside the home (at restaurants or school). The addition of certain seasonings and condiments can impact the final nutrient content of foods and the use of herbs and spices has become a target for healthy cooking interventions [39].

The category “food media” specifies how children were exposed to images of foods during the day. Food media activities ranged from the child taking a photo of foods they were eating to watching a food show, visiting a food-related website or reading a cookbook. Food advertisements were not included as they were not identified in images during preliminary review. Increased knowledge of how this sample employed food media may reveal targets for interventions utilizing new technology to promote food preparation in youth [40–42].

The category “meal related tasks” specifies non-food preparation behaviors that are relevant to meal times, such as clearing dishes or getting drinks. While slightly outside of “food preparation” as a concept, these activities may be relevant to understanding the range of behaviors that can be captured through questionnaires examining how children help with meals.

“Prep work” and “Cooking” categories include actual food preparation behaviors demonstrated by the sample and range from washing vegetables to using the stove. These categories classify instances where basic culinary ability is demonstrated with regard to both cold and hot preparations. Adolescents who were classified as demonstrating any of these behaviors were deemed to have demonstrated cooking skills and food preparation habits. Reliable assessment of these behaviors enables the determination of successful transmission of skills from youth-focused cooking classes to the home food environment.

“Observing” specifies the child witnessing the food preparation behaviors of others including adults or peers. This category is important since most food skills are likely transmitted within the home [43]. Modeling positive food behaviors and involving children while cooking are considered powerful parenting tools for encouraging healthy eating [44].

### Data analysis

The first author generated a basic codebook of behaviors, including definitions and examples, to capture the non-consumption food related behaviors of the participants in this project based on prior research on food preparation [45] and a preliminary review of the images. Codes were kept broad to accommodate the range of activities and limited culinary literacy demonstrated by the sample. During the preliminary review, codes were added if participants demonstrated previously uncategorized behaviors to ensure all food preparation activities demonstrated were captured. Once the codebook was developed, 20 % of the final total sample was coded by a second coder and inter coder reliability assessed using Cohen’s Kappa. After the initial double coding, inconsistencies were resolved through discussion between the two coders. Final categories were clarified (broadened or made more specific) and recoding was completed and analyzed to reflect the refined behavior classifications. Parent-reported participant demographics were assessed using descriptive statistics. Contingency tables were generated for age groups (9–11) and (12–13) to explore age-related trends in food/meal behavior.

## Results

### Participants

Forty-one participants provided image data for at least one day across the two studies. Data from 10 participants was excluded. Exclusions were made if the resulting images could not be analyzed using the activity software developed for the eButton, i.e. missing files (*n* = 2), software malfunction (*n* = 6) or file storage issues (*n* = 2). Analysis was completed on data for 31 participants. Each day of images took approximately one to two hours to code. Demographics of participants are shown in Table 2.

**Table 2:** Participant Demographics (*N* = 31)

Age	N	%
9	3	9.7
10	9	29.0
11	6	19.4
12	6	19.4
13	7	22.6
Gender		
Male	15	48.4
Female	16	51.6
Household Highest Education		
< High School Grad	2	6.5
High School Grad	1	3.2
Some College	4	12.9
College Graduate	6	19.4
Post Graduate	16	51.6
Annual Reported Income (USD)		
20,000 to 39,999	3	9.7
40,000 to 59,999	5	16.1
60,000 to 79,999	6	19.4
80,000 to 100,000	3	9.7
Over 100,000	12	38.7

One set of participants (*n* = 25) included all day camera images for two different days. The other set of participants (*n* = 6) provided only one day of images. Given the exploratory nature of this study, one day per participant was used for analysis. To address possible differences in the days selected for inclusion, day one and day two images were coded and compared for eleven participants. Frequency of overall food preparation behaviors and meal related tasks across the two days matched 81.4% of the time, although this mainly indicated that participants were similarly uninvolved in food preparation on both days. The number of demonstrated behaviors were similar, as those who did three or fewer behaviors (any activities) on day one, did three or fewer behaviors on day two 66.7% of the time. Those who demonstrated four or more behaviors (any activities) on day one, did four or more behaviors on day two 87.5% of the time.

Reliability for the recorded observations (yes/no for the day per individual behavior) was measured between two independent coders. Overall percent agreement between the two coders across all subcategories was high (agreement = 89.1%), with moderate/high inter-coder reliability (Cohen’s Kappa = .667). The main category disagreement centered around “observing”, specifically observing the food preparation of adults and food selection by peers. This category may need to be further refined with more specific definitions given that a wide spectrum of preparation and selection behaviors exist.

### Activities by age group (Table 3)

Observed activities are reported by age group in Table 3 (details of these behaviors are available in Table 1), given the potential variability in food preparation behavior between older and younger groups [46]. Each behavior was coded as either exhibited or not exhibited, regardless of the number of times demonstrated. The most common activities among the entire group included browsing in the pantry (51.6%) or fridge (71.0%). With regard to meal related tasks, getting drinks (58.1%) and clearing/washing dishes (71.0%) were the most common in the sample. Less than half of participants demonstrated food preparation work, with the most common activities being combining two or more ingredients (45.2%), and unwrapping of food packages (41.9%) while actual cutting or measuring ingredients was less common (12.9% each). Just over half the sample witnessed food preparation by adults according to the eButton image data (51.6%). Using a heat source to cook was uncommon in this sample, with the most common cooking method being microwaving (22.6%).

**Table 3:** Food/meal related activities by age (*N* = 31)

Activity	Age range		
	9 to 11^a^	12 to 13^b^	Ttl (%)^c^
Sample Size	18 (100%)	13 (100%)	31 (100%)
Browsing			
Pantry	7 (38.9)	9 (69.2)	16 (51.6)
Fridge	11 (61.6)	11 (84.6)	22 (71)
Grocery Store	2 (11.1)	0 (0)	2 (6.5)
Convenience Store or Café Counter	4 (22.2)	1 (7.7)	5 (16.1)
Kitchen Counter	3 (16.7)	2 (15.4)	5 (16.1)
At School	3 (16.7)	0 (0)	3 (9.7)
Altering Food on Plate/Adding Seasoning	4 (22.2)	3 (23.1)	7 (22.6)
Food Media, Recipes, Cookbooks, Photo	0 (0)	3 (23.1)	3 (9.7)
Tasks			
Plating	3 (16.7)	6 (46.2)	9 (29)
Getting Drinks	10 (55.6)	8 (61.5)	18 (58.1)
Clearing and/or washing dishes	13 (72.2)	9 (69.2)	22 (71)
Setting the Table	1 (5.6)	0 (0)	1 (3.2)
Prep Work			
Washing and/or Peeling Produce	0 (0)	4 (30.8)	4 (12.9)
Cutting	2 (11.1)	2 (15.4)	4 (12.9)
Cracking Eggs	0 (0)	1 (7.7)	1 (3.2)
Combining 2+ ingredients by hand	8 (44.4)	6 (46.2)	14 (45.2)
Measuring	1 (5.6)	3 (23.1)	4 (12.9)
Unwrapping	7 (38.9)	6 (46.2)	13 (41.9)
Using Blender, Mixer, Small appliance	0 (0)	1 (7.7)	1 (3.2)
Observing			
Food Selection by Adults	0 (0)	4 (30.8)	4 (12.9)
Food Selection by Peers	3 (16.7)	2 (15.4)	5 (16.1)
Any Food Preparation by Adults	9 (50.0)	7 (53.8)	16 (51.6)
Any Food Preparation by Peers	0 (0)	2 (15.4)	2 (6.5)
Cooking			
Stove	3 (16.7)	2 (15.4)	5 (16.1)
Oven (Baking)	0 (0)	1 (7.7)	1 (3.2)
Microwave	3 (16.7)	4 (30.8)	7 (22.6)

Table 3: Food/meal related activities by age. Table showing frequencies of activity by major and sub category. Totals indicate number of children who demonstrated each individual behavior at least one time through the course of a day

^a^= # of participants between the ages of 9–11 demonstrating behavior (# participants/total number of 9–11 year olds (*n* = 18))

^b^= # of participants between the ages of 12–13 demonstrating behavior (# participants/total number of 12–13 year olds (*n* = 13))

^c^= total # of participants demonstrating behavior in all age groups (# participants/total sample (*n* = 31))

No statistically significant differences were found between the older and younger age groups with regard to food preparation behaviors, possibly due to the small sample size. Post hoc power analysis shows variable power depending on activity observed, ranging from very low (3.1%) to moderately-low (70.4%) power. However, some slight differences between the groups were identified. In this sample, younger children browsed for foods at school more often than the older group (16.7% vs 0%). The older group demonstrated more use of food media (23.1% vs 0%), more meal plating (46.2% vs 16.7%) and more observation of food selection by adults (30.8% vs 0%) than the younger group. The older participants also showed more food preparation behaviors than the younger group with regard to washing/peeling produce (30.8% vs 0%) and measuring ingredients (23.1% vs 5.6%).

Approximately one to two hours per participant was required to process images, and identify and classify food activity behaviors. Technical difficulties with the software developed for the eButton caused delays and some photo sets were not usable. The software was still in early stages and therefore updated on a regular basis, making later analysis more time-effective as issues were resolved with developers.

## Discussion

Food preparation and related activities were consistently identifiable using eButton all-day images in this sample of pre- and early adolescents. Participants were frequently seen browsing for food around their homes, but were less frequently seen actually preparing foods for cooking or cooking foods themselves. The most common food preparation activities were unwrapping, combining two or more ingredients, and microwaving, indicating participants were more likely to work with pre-packaged foods when at home as opposed to meals made from scratch. When food preparation and related activities did occur, they were clearly identifiable from the angle of the eButton camera. Further, the eButton activity software allowed for efficient classification of food preparation activities, as the software automatically segmented photos into homogenous events.

Previous research has explored all day camera and other image-generating technologies to measure food intake and physical activity [47–49]. Most such studies validated their method through concordance with a reference intake measure. A systematic review of the new technologies concluded these tools supported adult self-report data by giving more information about underreported foods [13]. The authors noted, however, the importance of supporting images with information about food preparation including ingredients and cooking method [13]. Our findings suggest such information can be gleaned from passive all-day camera data, which may support dietary assessments.

Research on food preparation among 4746 adolescents found 49.8% assisted with food shopping [46]. Our sample showed 6.5% of participants had images that appeared to be in grocery stores or markets on the day they wore the eButton. One reason for the discrepancy may be the sample size/selection, or the broader timeframe of the original study, which consisted of a single item: “In the past week, how many times did you help shop for groceries?”. Most participants in the original study only helped with grocery shopping a maximum of one time per week (82.2%) [46]. Thus, all day cameras need to be worn for an entire week or longer to obtain accurate data on this behavior consistent with the questionnaire.

In the same survey study, a high percentage of adolescents reported helping with dinner (68.6%), based on a single item: “In the past week, how many times did you help prepare food for dinner?” [46]. This item is open to broad interpretation. Our results show that while most participants did at least one food or meal related activity during the day, they demonstrated little actual food preparation. Camera technology may enhance self-report studies by identifying what adolescents are referring to when they report helping with meals, or help improve the specification of behaviors targeted in study questionnaires.

Another survey study of 2029 Minnesota adolescents used a similar general frequency of food preparation assessment as above, and found 42.2% of girls and 28.4% of boys reported helping prepare meals at least 3 times per week [23]. Future studies with imaging technology using larger sample sizes should consider examining food and meal behaviors by sex over longer periods of time.

A smaller survey of 289 African American adolescents in Maryland used a similar frequency item: “In the past 7 days, how often did you prepare food for yourself or others (including making yourself lunch)?”, followed with space to report foods prepared and preparation method (classified as fried, baked, microwaved, not cooked, or other). While most participants reported some food preparation, the most commonly noted food was cereal, with noodles and sandwiches also being widely reported. The most common food preparation method was “not cooked” followed by “microwaved” [50]. These findings are consistent with our data. Sandwiches, cereal and instant variety noodles are essentially prepared by combining two ingredients, an activity which was demonstrated by 45.2% of our sample. Of the heat based cooking methods used, microwaving was the most common in our study (22.6%). Thus, while adolescents are indeed helping prepare meals, they may not be demonstrating cooking skills beyond those needed for convenience foods.

The inactivity of adolescents in regard to more complex food preparations highlights the need for cooking skill interventions in youth. The coding system developed for this study is unique in that it encompasses details of food preparation. This system could be further expanded to assess the duration and quality of food preparation in response to a cooking intervention. Such an evaluation tool could identify the frequency of using intervention resources (e.g. recipe books) in the home and/or reflect an evaluation scale of positive (e.g. reducing added sugars) to more negative (e.g. deep frying) food preparation behaviors [45].

This is the first study to use all day camera images to classify food preparation and meal related behaviors in a sample of pre- and early adolescents. When food preparation behaviors were identified, camera angles offered the ability to clearly see behaviors in action. A wide range of behavior classifications allowed for details to be gathered well beyond standard self-report assessments of food preparation behavior in youth. Our findings support the use of image-based technology in food preparation intervention evaluations targeting adolescents.

Limitations include the small volunteer sample and reliance on secondary analysis. Single day images are also a limitation, as the recorded day may not be representative of normal behavior. The lack of a reference assessment method, such as direct observation, with which to compare both eButton and self reported values, limits our ability to say whether the eButton provides a better method than self report. A subsequent study is in progress to compare eButton images with direct observation and self-report assessments of food preparation events. Reactivity bias is also a potential issue, as participants may have altered behavior due to the knowledge that they were being recorded. However, previous research has shown acceptability and feasibility of using this technology for dietary assessment in adolescents [15]. Although the eButton camera angle is wider than most cameras utilized in smartphones (120° angle of view), it may still miss certain scenes of interest. Food advertisement exposure, for example, was not commonly seen in the sample, potentially due to the downward angle of the camera and four second interval between images. This angle appears to capture extended mobile phone activity, book/magazine reading and most television, but not billboards, posters or restaurant signage. In addition, the camera has a fixed orientation with respect to the vertical line (the line orthogonal to the surface of the earth). The orientation may not be optimal for every child because of variability in body height. These technical issues need to be investigated further to improve device performance in a food preparation study. Further, 24% of our data could not be analyzed due to issues with the software. The activity categorization software is still in a nascent stage, and therefore software updates will be continually needed to ensure all data collected can be processed.

Future research can build on this study by integrating self-report questions regarding food preparation into study design to better understand the relationship between self-reported and actual behaviors. Audio information may also be important, and was not available in the version of the eButton device used in this study. Audio would be useful to understand how a child responds when being taught or helping during active food preparation. Analysis of the camera images was time consuming, requiring one to two hours per participant per day of images. This type of work will benefit from automating all day image processing, and such automation software is currently in development at the University of Pittsburg [14]. Once automated to identify eating events, researchers could target images just before consumption to identify food preparation events more quickly and code food preparation practices. This would allow for more detailed analysis of cooking behaviors, while reducing the overall amount of data for analysis. Thus, all day cameras may be valuable evaluation tools for nutrition education programs, especially those that include food preparation skill development.

## Conclusions

Youth cooking programs have gained in popularity but it is unclear if children are cooking at home or otherwise learning positive food or meal related behaviors. Although children who “help” prepare meals may obtain some dietary benefit, most relevant measurement tools are questionnaires that rely on simple single items (i.e.: Did you help make dinner?). Self-report questionnaires, while useful, offer limited detail and unknown validity regarding preparation practices. The eButton shows promise to identify food preparation behaviors in pre- and early adolescents. More work integrating this technology into nutrition intervention evaluations for adolescents and other populations is needed.

## Abbreviations

HEI: Healthy Eating Index
STROBE: Strengthen the Reporting of Observational Studies in Epidemiology
